# Cigarette Prices and Smoking Prevalence After a Tobacco Tax Increase — Turkey, 2008 and 2012

**Published:** 2014-05-30

**Authors:** Deliana Kostova, Linda Andes, Toker Erguder, Ayda Yurekli, Bekir Keskinkılıç, Sertaç Polat, Gönül Çulha, Evin Aras Kilinç, Enver Taştı, Yılmaz Erşahin, Mehmet Özmen, Ramazan San, Hilal Özcebe, Nazmi Bilir, Samira Asma

**Affiliations:** 1 Office on Smoking and Health, National Center for Chronic Disease Prevention and Health Promotion, CDC; 2 Prevention of Noncommunicable Diseases, World Health Organization, Geneva, Switzerland; 3 Turkey Ministry of Health, Ankara, Turkey; 4 Turkish Statistical Institute, Ankara, Turkey; 5 Institute of Public Health, Hacettepe University, Ankara, Turkey

Raising the price of tobacco products has been shown to reduce tobacco consumption in the United States and other high-income countries, and evidence of this impact has been growing for low- and middle-income countries as well (1,2). Turkey is a middle-income country surveyed by the Global Adult Tobacco Survey (GATS) twice in a 4-year period, in 2008 and 2012. During this time, the country introduced a policy raising its Special Consumption Tax on Tobacco and implemented a comprehensive tobacco control program banning smoking in public places, banning advertising, and introducing graphic health warnings. The higher tobacco tax took effect in early 2010, allowing sufficient time for subsequent changes in prices and smoking to be observed by the time of the 2012 GATS. This report uses data from GATS Turkey to examine how cigarette prices changed after the 2010 tax increase, describe the temporally associated changes in smoking prevalence, and learn whether this smoking prevalence changed more in some demographic groups than others. From 2008 to 2012, the average price paid for cigarettes increased by 42.1%, cigarettes became less affordable, and smoking prevalence decreased by 14.6% (Figure). The largest reduction in smoking was observed among persons with lower socioeconomic status (SES), highlighting the potential role of tax policy in reducing health disparities across socioeconomic groups.

GATS is an ongoing, nationally representative household survey of noninstitutionalized adults aged ≥15 years. The survey uses a multistage geographically clustered sample design. The indicators described in this report were obtained by summarizing the individual responses of participants in GATS Turkey 2008 (9,030 completed interviews) and 2012 (9,851 completed interviews). Response rates for GATS Turkey were 90.1% in 2012 and 93.7% in 2008. Smoking prevalence estimates were based on self-reported current smoking, which included both daily and less-than-daily smoking. Prices paid per 20 cigarettes were calculated from the responses of current smokers of manufactured cigarettes, which provide data on amounts spent and quantities purchased during the most recent cigarette purchase. Price indicators for 2008 were adjusted for inflation to be comparable with 2012 values. The cigarette price indicators in this report are not brand-specific, but represent the average amount spent per 20 cigarettes across the range of brand choices in each year. The examined indicators and their relative change from 2008 to 2012 were stratified by demographic characteristics including sex, age, urbanicity, education, and wealth. The wealth index category for each respondent was created based on self-reported ownership of certain core household items in GATS (3).

Changes in cigarette affordability during the study period were evaluated using the relative-income price of cigarettes, which represents prices adjusted for country income level (4). The relative-income price was calculated as the ratio of the average price paid per 2,000 cigarettes in each GATS year to that year’s gross domestic product (GDP) per capita (4).

After adjusting for inflation, the average real price paid per 20 cigarettes in Turkey increased by 42.1% during 2008–2012, from 4.0 to 5.7 Turkish lira (Table 1). The increase in the purchasing price varied across demographic groups; for instance, it was estimated to be smaller among younger smokers and among smokers with less wealth. As the cost of cigarettes increased, the average smoking rate dropped by 14.6% during 2008–2012, from 30.1% to 25.7% (Table 2). The largest decrease in smoking occurred among persons of lower SES, who were in the lowest wealth and education categories (Table 2). The relative reduction in smoking among those in the bottom tercile of the wealth index (−30.3%) was twice as large as among those in the middle wealth tercile (−13.9%), and nearly three times larger than among those in the top wealth tercile (−11.1%).

On average, cigarettes in Turkey became less affordable during 2008–2012. Cigarette affordability, represented by the relative-income price, falls when the growth in cigarette prices outpaces the growth in GDP per capita. The relative-income price of cigarettes in Turkey increased by approximately 30% from 2008 to 2012 (Table 1), indicating that during this period cigarette prices in Turkey increased faster than the country’s per capita income, corresponding to a significant reduction in affordability.

## Discussion

After the 2010 increase in tobacco taxes in Turkey, the average price paid for cigarettes increased, cigarettes became less affordable, and a statistically significant drop in smoking rates occurred. The reduction in smoking was substantially larger among persons with lower SES. These findings document the presence of an inverse relationship between cigarette prices and smoking in Turkey, and confirm previous analytic findings that this relationship is especially strong in lower-income populations (5). This underscores the potential of a tobacco price increase to reduce tobacco use and to help reduce health disparities by lowering smoking prevalence at a higher rate in vulnerable populations.

Although the average purchasing price of cigarettes increased for all demographic groups, it increased at a slightly lower rate among smokers in the lowest wealth tercile than among those at the middle or higher ends of the wealth spectrum. Similarly, younger smokers experienced a smaller increase in the average purchasing price than older smokers. These demographic differences indicate that smokers who are younger or low-income might be more likely to engage in price-minimizing behavior when facing a tax increase (examples of such behavior include switching to less expensive brands and buying in bulk).

The demographic breakdown of the 2008–2012 changes in purchasing price and smoking rates in Turkey shows that groups with relatively tighter income constraints, such as young adults or persons with a lower wealth index, reported smaller increases in cigarette prices paid and at the same time experienced the steepest declines in smoking prevalence. These findings have implications with respect to tax regressivity. An existing tax is regressive if it imposes a greater burden, relative to income, on those with lower wealth. However, a tobacco tax increase does not have regressivity characteristics. The increase in tobacco tax in Turkey was associated with a greater reduction in smoking among persons with the lowest wealth than among wealthier persons. This suggests that the tax increase did not have a regressive outcome, because smoking and its associated expense declined most among those who could least afford the habit.

Comparing the change in the average purchasing price of cigarettes with the concurrent increase in cigarette tax can help infer the tax pass-through, which is the extent to which the tax increase was reflected in the final consumer price. A full pass-through of the tax onto the final price is more likely to influence consumption than a partial pass-through, where the tax increase is partially absorbed by the producer and might not fully reach the consumer. In the case of Turkey, the tax pass-through appears to be complete, optimizing the potential tax impact. The cigarette tax level in Turkey, measured as the share of total tax to retail price, rose from 0.74 to 0.80 during 2008–2012 (Turkey Ministry of Finance, General Directorate of Revenue Policies, unpublished data, 2013). Applying these tax shares to the average cigarette price paid in each year, and comparing the change in the average tax amount with the change in the average price, it is estimated that the pass-through of the 2010 tax increase in Turkey was more than one-to-one. This indicates that the tax increase might have been accompanied by an additional price increase from the producer side, timed to coincide with the tax change. A producer-initiated price increase that shadows a concurrent tax increase is a common pricing strategy in some markets. In markets perceived as tobacco use strongholds, such as Turkey, price increases that further augment the tax pass-through would be driven by a tobacco producer’s anticipation of higher profits. This is in contrast to the United States, where a recent shift has occurred toward pricing strategies that reduce the tax pass-through, such as tobacco industry offering of discounts and coupons, which limit the full potential of taxes for reducing consumption (6).

The findings in this report are subject to at least three limitations. First, the examined indicators are based on self-reported individual answers to survey questions and therefore are subject to recall bias. Second, because of small sample sizes, important differences across demographic groups within each year sample might not be statistically significant. This affects especially the demographic breakdown of cigarette prices paid, which are based on a subsample of smokers. Finally, these data do not establish cause-and-effect relationships because of the observational nature of the report, which does not control for other tobacco control measures introduced at the same time as the tax increase.

Turkey’s smoking rates historically have been among the world’s highest. This report describes a considerable shift in smoking behavior, occurring even when the baseline levels of tobacco use and addiction in the population are relatively high. Turkey’s experience with cigarette price change might be informative to policymakers in other low- and middle-income countries, where the majority of tobacco-related deaths are expected to occur in the near future (7).

What is already known on this topic?

There is increasing evidence that raising the prices of tobacco products can reduce tobacco use in low- and middle-income countries, where most of the global tobacco-related disease burden is expected to occur. Turkey is a middle-income country with smoking rates that historically have been among the world’s highest. In 2010, Turkey increased its Special Consumption Tax on Tobacco, increasing the price of cigarettes.

What is added by this report?

After the increase in tobacco tax, the average price paid for cigarettes in Turkey increased by 42% during 2008–2012, cigarettes became less affordable, and the average smoking prevalence declined by 15%. The largest reduction in smoking prevalence (30% relative change from 2008 to 2012) was observed among persons with the lowest socioeconomic status.

What are the implications for public health practice?

These survey results establish a link between a tobacco price increase and a decline in tobacco use, and show the potential of tobacco taxes and prices to help reduce health disparities by lowering smoking prevalence at a higher rate in vulnerable populations.

## Figures and Tables

**FIGURE:**
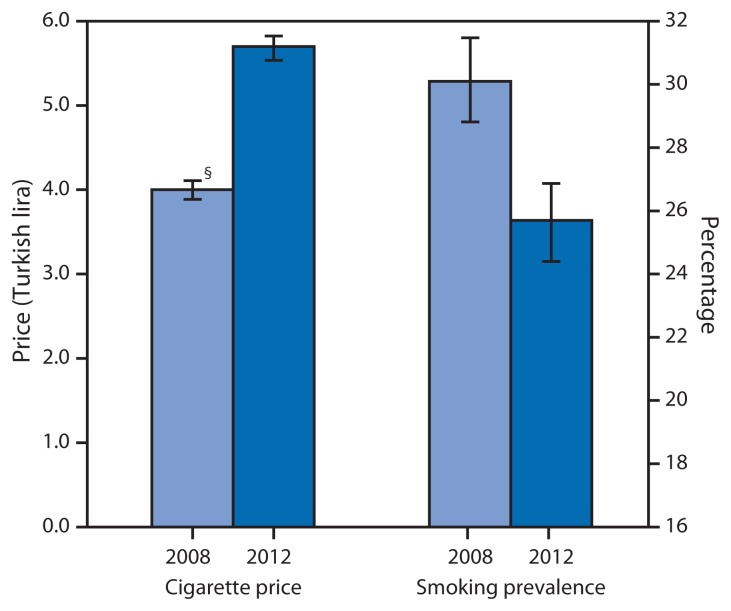
Average cigarette prices* (in Turkish lira) and smoking prevalence^†^ — Global Adult Tobacco Survey, Turkey, 2008 and 2012 * Average price paid per 20 manufactured cigarettes in constant 2012 Turkish lira. ^†^ Prevalence of current smoking of manufactured cigarettes. ^§^ 95% confidence interval.

**TABLE 1:** Average price (in Turkish lira) paid per 20 manufactured cigarettes, by selected demographic characteristics — Global Adult Tobacco Survey, Turkey, 2008 and 2012

	2008		2008 (inflation adjusted)*		2012		Relative change from 2008 (inflation adjusted)* to 2012	
				
Characteristic	Price	(95% CI)	Price	(95% CI)	Price	(95% CI)	%	(95% CI)
**Overall**	**3.3**	**(3.2–3.3)**	**4.0**	**(3.9–4.1)**	**5.7**	**(5.5–5.8)**	**42.1**	**(38.0–46.2)** †
**Sex**								
Male	3.3	(3.2–3.4)	4.0	(3.9–4.1)	5.7	(5.6–5.9)	42.1	(37.7–46.5)†
Female	3.1	(3.0–3.2)	3.8	(3.6–3.9)	5.4	(5.2–5.6)	43.9	(36.1–51.6)†
**Age group (yrs)**								
15–24	3.4	(3.2–3.5)	4.1	(3.9–4.3)	5.6	(5.3–5.9)	35.7	(26.4–45.1)†
25–44	3.4	(3.3–3.4)	4.1	(4.0–4.2)	5.7	(5.5–5.8)	38.5	(33.8–43.2)†
45–64	3.1	(3.0–3.2)	3.8	(3.6–3.9)	5.7	(5.5–6.0)	52.2	(44.2–60.3)†
≥65	2.7	(2.5–2.9)	3.3	(3.1–3.5)	5.2	(4.6–5.8)	57.7	(37.1–78.4)†
**Residence**								
Urban	3.3	(3.3–3.4)	4.1	(4.0–4.2)	5.7	(5.6–5.9)	40.7	(35.9–45.5)†
Rural	3.1	(3.0–3.2)	3.8	(3.6–3.9)	5.5	(5.3–5.6)	44.7	(37.8–51.7)†
**Education**								
Not graduated	2.8	(2.6–3.0)	3.4	(3.2–3.7)	4.8	(4.3–5.4)	39.7	(21.7–57.6)†
Primary	3.1	(3.0–3.2)	3.8	(3.7–3.9)	5.5	(5.3–5.7)	43.9	(37.8–49.9)†
Secondary	3.3	(3.1–3.4)	4.0	(3.8–4.2)	5.6	(5.4–5.8)	40.0	(32.5–47.6)†
High school	3.5	(3.4–3.6)	4.3	(4.2–4.5)	5.9	(5.7–6.2)	37.2	(30.3–44.1)†
University or higher	3.6	(3.4–3.8)	4.4	(4.2–4.7)	6.2	(5.9–6.5)	39.8	(30.4–49.3)†
**Wealth index**								
Bottom tercile	2.9	(2.8–3.0)	3.6	(3.4–3.7)	4.9	(4.6–5.2)	38.3	(28.2–48.4)†
Middle tercile	3.2	(3.2–3.3)	4.0	(3.9–4.1)	5.5	(5.3–5.7)	39.1	(33.7–44.5)†
Top tercile	3.6	(3.4–3.7)	4.3	(4.2–4.5)	6.1	(5.9–6.2)	39.5	(33.5–45.6)†
**Unweighted no. of current smokers of manufactured cigarettes**			2,384		2,218			
**Affordability index (relative income price) (%)** §			2.4		3.0		29.9†	

**Abbreviation:** CI = confidence interval.

* 2008 values were adjusted for inflation to represent constant 2012 Turkish lira.

† Statistically significant.

§ Calculated as the ratio of the average price paid per 2000 cigarettes to gross domestic product per capita.

**TABLE 2:** Prevalence of current manufactured cigarette smoking, by selected demographic characteristics — Global Adult Tobacco Survey, Turkey, 2008 and 2012

	2008		2012		Relative change from 2008 to 2012	
			
Characteristic	%	(95% CI)	%	(95% CI)	%	(95% CI)
**Overall**	**30.1**	**(28.8–31.4)**	**25.7**	**(24.5–27.0)**	−**14.6**	**(**−**20.1 to** −9.0)*
**Sex**						
Male	45.8	(43.7–47.9)	39.2	(37.2–41.3)	−14.4	(−20.3 to −8.5)*
Female	14.9	(13.8–16.2)	12.6	(11.5–13.8)	−15.7	(−26.0 to −5.4)*
**Age group (yrs)**						
15–24	24.5	(21.5–27.8)	19.1	(16.6–22.0)	−22.0	(−36.8 to −7.2)*
25–44	38.8	(36.8–40.7)	34.4	(32.5–36.2)	−11.3	(−17.8 to −4.8)*
45–64	27.9	(25.9–30.1)	23.8	(21.8–25.9)	−14.8	(−24.5 to −5.2)*
≥65	9.2	(7.5–11.3)	8.0	(6.4–9.8)	−13.7	(−39.0 to 11.7)
**Residence**						
Urban	32.4	(30.7–34.0)	27.8	(26.2–29.4)	−14.1	(−20.7 to −7.6)*
Rural	24.8	(23.0–26.8)	20.3	(18.7–22.1)	−18.2	(−27.3 to −9.0)*
**Education**						
Not graduated	13.1	(10.7–16.0)	9.5	(7.7–11.8)	−27.2	(−48.6 to −5.8)*
Primary	32.7	(30.7–34.8)	27.8	(25.8–29.8)	−15.1	(−23.2 to −7.0)*
Secondary	30.3	(27.1–33.6)	26.0	(23.5–28.6)	−14.2	(−26.6 to −1.8)*
High school	39.9	(36.6–43.2)	32.7	(29.9–35.6)	−18.0	(−27.7 to −8.2)*
University or higher	31.3	(27.6–35.3)	26.5	(23.2–30.1)	−15.2	(−30.3 to −0.1)*
**Wealth index**						
Bottom tercile	25.7	(23.2–28.4)	17.9	(15.4–20.7)	−30.3	(−42.6 to −17.9)*
Middle tercile	31.9	(30.3–33.7)	27.5	(25.9–29.2)	−13.9	(−20.7 to −7.0)*
Top tercile	29.6	(27.2–32.0)	26.3	(24.5–28.1)	−11.1	(−20.4 to −1.7)*
**Unweighted no. of respondents (total)**	**9,030**		**9,851**			

**Abbreviation:** CI = confidence interval.

* Statistically significant.
